# Exploration and Evaluation of Adverse Drug Reactions Documented in a Tertiary-Care Hospital in Chennai: An In-Depth Retrospective Observational Study

**DOI:** 10.7759/cureus.60977

**Published:** 2024-05-24

**Authors:** Preetha Selva, Sheela Durairajan

**Affiliations:** 1 Department of Pharmacology, Saveetha Medical College and Hospital, Saveetha Institute of Medical and Technical Sciences, Saveetha University, Chennai, IND

**Keywords:** type of reactions, severity assessment scale, pharmacovigilance, causality assessment scale, antibiotics, adverse drug reactions

## Abstract

Introduction: While drugs are intended to benefit patients, adverse drug reactions (ADRs) represent a significant negative outcome of drug consumption. They rank as the sixth leading cause of death among hospitalized patients. Many harmful effects are preventable and can reduce morbidity, mortality, and hospitalization duration. This study is a valuable resource for physicians, aiding in the safe and optimal use of medications.

Methodology: This retrospective observational study, conducted at the Pharmacovigilance Center of Saveetha Medical College and Hospital, Chennai, India, received approval from the Institutional Ethics Committee. All adverse drug interactions reported in our hospital from January 2019 to February 2024 were included after screening for inclusion and exclusion criteria. The collected reactions were analyzed, assessed, and evaluated between February 2024 and April 2024. Data on the drugs causing adverse reactions, the types of reactions, and the treatments administered were collected and documented. The reactions were categorized using the Rawlins and Thompson classification, while causality and severity were assessed using the standard Naranjo causality and modified Hartwig and Siegel severity assessment scales.

Results: During the study, 252 ADRs were documented by the Central Drugs Standard Control Organization. The gender distribution showed 123 cases (48.8%) in males and 129 cases (51.2%) in females, with a higher prevalence in the 20-40 age group. The departmentwise distribution revealed the highest number of ADRs in Obstetrics and Gynecology (60 cases, 24%), followed by General Surgery (52 cases, 21%), General Medicine (44 cases, 17%), Pediatrics (22 cases, 9%), and Emergency Medicine (20 cases, 8%).

Antimicrobial drugs constituted the majority of ADRs (149 cases, 59.1%), followed by vitamins and mineral supplements (21 cases, 13.8%), contrast dye (15 cases, 6%), antituberculosis drugs (15 cases, 6%), analgesics (13 cases, 5.2%), and gastrointestinal (GIT) drugs (8 cases, 3.2%). Cefotaxime was the most commonly reported antibiotic (42 cases, 28.2%), followed by Ciprofloxacin (41 cases, 27.5%). Among vitamins and mineral supplements, iron sucrose was implicated in the highest number of ADRs (15 cases, 71.4%).

The parenteral route of drug administration showed the highest incidence of ADRs (229 cases, 91%), followed by oral (20 cases, 8%) and topical routes (3 cases, 1%). Dermatological manifestations were most frequently reported (196 cases, 77.8%), followed by GIT symptoms (27 cases, 10.7%), and other manifestations such as shivering, fever, seizures, and dyspnea (29 cases, 11.5%).

Based on the Naranjo causality assessment scale, 179 ADRs (71%) were categorized as probable, 55 (22%) as possible, 10 (4%) as certain, and 8 (3%) as doubtful. Approximately 47.2% of ADRs were self-limiting, while 44.1% required symptomatic treatment and 8.7% necessitated aggressive treatment, leading to a prolonged hospital stay or admission to the intensive care unit.

Conclusion: The pattern of ADRs in our hospital aligns with findings from other studies. While many of these reactions are unpredictable and mild, they underscore the importance of raising awareness among clinicians and regulatory authorities to promote safe medication use and prevent potentially serious outcomes.

## Introduction

An adverse drug reaction (ADR) is defined by the WHO as “any noxious, unintended, or undesired effect of a drug that occurs at doses used in humans for prophylaxis, diagnosis, therapy, or modification of physiological functions” [1]. Though drugs are used for various benefits for patients, ADRs are an important negative consequence of consuming drugs. ADRs comprise the sixth most prominent reason for death, directly or indirectly, among patients admitted to the hospital [2]. Around 10%-20% of patients admitted to the hospital develop ADRs, according to earlier studies [3-5]. The use of multiple medications resulting in drug-drug interactions, numerous comorbidities in the patient, frequent evolution of drastic illnesses, and inappropriate medications are the most common causes of ADRs. These reactions badly affect the patient’s quality of life, increase the duration of stay in the hospital and hospital visits, increase the cost incurred for the patient, and can also result in death [6]. To prevent such ADRs, the Central Drugs Standard Control Organization (CDSCO), supported by the Government of India, the Ministry of Health and Family Welfare, and the Pharmacovigilance Programme of India, has set up various ADR monitoring centers all over India. These centers enable effective vigilance of ADRs and assure the safe use of drugs. Despite the centers being organized, ADR reporting is still ineffective in resource-limited countries. Insufficient knowledge, a lack of proper training, and poor acquaintance among healthcare providers hinder the timely and proper reporting of ADRs [7]. Despite the above limitations, spontaneous reporting of ADRs is a mainstay for creating safety signals [8].

The significance of identifying potential risk factors for ADRs must be known to minimize preventable ADRs. The data regarding the drugs most frequently causing ADRs and their therapeutic category, the demographic details of patients who suffered from ADRs and concurrent medications used, and the assessment of ADRs based on causality and severity should be known to assist practitioners in minimizing, preventing, and managing ADRs more effectively.

In the present study, an attempt has been made to recognize and distinguish the pattern of ADRs reported in our hospital. The ADRs were assessed for causality and severity using standard assessment scales, such as the Naranjo causality assessment scale and modified Hartwig and Siegel assessment scales, respectively. This study will enable clinicians to identify frequently encountered ADRs in our hospital, minimize preventable ADRs, and assist them in handling ADRs more efficiently. Hence, the study will serve as a database, which will help physicians provide safe and optimum use of medicines.

## Materials and methods

Study design, duration, place, and sampling technique

This retrospective observational study was conducted in the pharmacovigilance center of Saveetha Medical College and Hospital, Chennai, India. All ADRs reported in our hospital from January 2019 to February 2024 were included in the study using convenience sampling after screening for inclusion and exclusion criteria. These reported reactions were analyzed, assessed, and evaluated from February 2024 to April 2024.

Study population

Inclusion Criteria

The study included all ADRs reported with comprehensive information, adhering to mandatory fields essential for a valid case report. This encompassed patient details (initials, age at onset of reaction), suspected adverse reaction, description of the reaction (including reaction terms), reaction date, and suspected medication, including both brand and generic names. Reports were submitted to the Pharmacovigilance Center of Saveetha Medical College and Hospital from January 2019 to February 2024.

Exclusion Criteria

Incomplete and illegible data were omitted from the study. Ineligible data encompass information unsuitable for inclusion in the form due to several reasons, such as incompleteness, irrelevance, inaccuracy, confidentiality, duplication, and being out-of-scope. Hence, we included only pertinent, precise, and comprehensive data in ADR forms to uphold the integrity and effectiveness of pharmacovigilance endeavors.

Procedure

The study was carried out after getting approval from the Institutional Ethics Committee with approval number SMC/IEC/2020/03/260. All ADRs reported in the pharmacovigilance center of our hospital by various departments during the study period were collected. The ADRs reported were categorized based on the patient's demographic details, gender, department, type of reaction, drug-causing ADR and their therapeutic class, organ system affected, and their management. The types of reactions reported were categorized using Rawlins and Thompson classifications as type A (expected or augmented) reactions and type B (bizarre or unexpected) reactions [9]. The causality of the reported ADRs was assessed using the standard Naranjo causality assessment scale, which consists of a set of 10 standard questions with scores of -1, 0, +1, or +2 assigned to each question based on answers of yes, no, or do not know [10]. The causal relationship of ADR is definite if the final score is 9 or higher, probable if 5 to 8, possible if 1 to 4, and doubtful if 0 or less. The severity of ADRs reported is assessed using modified Hartwig and Siegel severity assessment scales as mild, moderate, and severe [11]. Mild reactions include no treatment for ADR, changes in treatment of suspected drugs, and an increase in the duration of hospital stays. Moderate reactions include treatment for ADR, changes in the treatment of suspected drugs, and an increase in the duration of hospital stay by at least one day. Severe reactions include ADR requiring intensive medical care, causing permanent harm or damage, and resulting in death directly or indirectly for the patient.

Statistical analysis

The data were represented as frequency distribution tables. They were analyzed for independence by Pearson's chi-square test (χ^2^ test). For simplicity, the variables were grouped into two or three categories. A probability of 0.05 or less was considered statistically significant. SigmaPlot version 14.5 (Systat Software Inc., San Jose, USA) was used for statistical analysis. Descriptive statistics in the form of tables, charts, and percentages were used.

## Results

A total of 252 ADRs reported in our hospital during the study period were included in our study. All the ADRs were reported in CDSCOs' suspected ADRs reporting form. The yearwise distribution of reported ADRs is shown in Table 1.

**Table 1:** Yearwise distribution of reported ADRs ADRs: adverse drug reactions

Year	Number of ADRs reported
JAN 2019-DEC 2019	54 (21.42%)
JAN 2020-DEC 2020	33 (13.09%)
JAN 2021-DEC 2021	22 (8.73%)
JAN 2022-DEC 2022	54 (21.42%)
JAN 2023-DEC 2023	77 (30.55%)
JAN 2024-FEB 2024	12 (4.76%)

Of the 252 ADRs reported, 123 (48.8%) were in males and 129 (51.2%) were in females. ADRs were more common in the 20-40 age group. The demographic details of ADRs reported are represented in Table 2.

**Table 2:** Demographic details of reported ADRs ADRs: adverse drug reactions A chi-square test of independence was done to find out the relation between gender and ADRs reported. The chi-square statistic (χ^2^) is 0.2857. The p value is 0.59298. The result is not significant at p < 0.05. ^*^A chi-square test of independence was done to find out the relation between age and ADRs reported. The chi-square statistic (χ^2^) is 147.748. The p value is <0.00001. The result is statistically significant at p < 0.05.

Parameters	Number	Percentage (%)	Chi-square value	P value
Gender				
Male	123	48.8	0.2857	0.59298
Female	129	51.2		
Age (years)				
Less than 10	8	3.2	147.748	<0.00001^*^
10-20	34	13.5		
20-40	107	42.5		
40-60	70	27.8		
Above 60	33	13.1		

**Figure 1:**
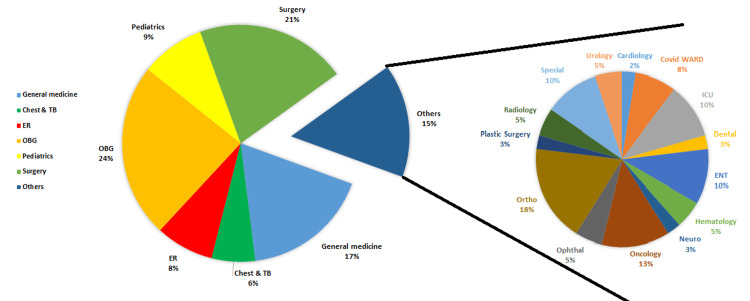
Departmentwise distribution of ADRs reported from 2019 to 2024 ADR: adverse drug reactions; OBG: Obstetrics and Gynecology; CHEST & TB: Chest and Tuberculosis; ER: Emergency Room; OPHTHAL: Ophthalmology; ORTHO: Orthopedics (the branch of medicine dealing with the correction of deformities of bones or muscles); NEURO: Neurology; ICU: intensive care unit; ENT: Ear, Nose, and Throat

The departmentwise distribution of ADRs is represented in Figure 1. Maximum ADRs were reported from the Obstetrics and Gynecology Department 60 (24%), followed by General Surgery 52 (21%), General Medicine Departments 44 (17%), Pediatrics 22 (9%), and the Emergency Medicine Department 20 (8%).

According to Rawlins and Thompson classification, around 81 (32%) of the reactions were type A, and 171 (68%) were type B.

**Figure 2:**
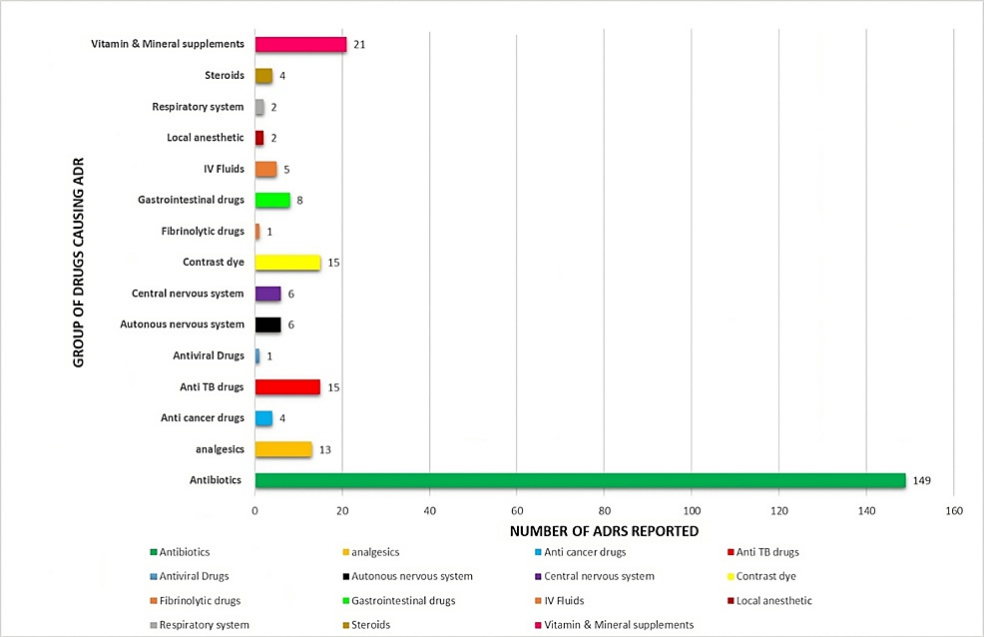
Group of drugs causing ADRs ADR: adverse drug reaction; IV fluids: intravenous fluids; anti-TB drugs: antituberculosis drugs

Antibiotics were the most common group of drugs causing ADRs 149 (59.1%) followed by vitamins and mineral supplements 21 (13.8%), contrast dye 15 (6%), antituberculosis (anti-TB) drugs 15 (6%), analgesics 13 (5.2%), and gastrointestinal (GIT) drugs 8 (3.2%). The number of ADRs reported in each drug group is represented in Figure 2.

**Figure 3:**
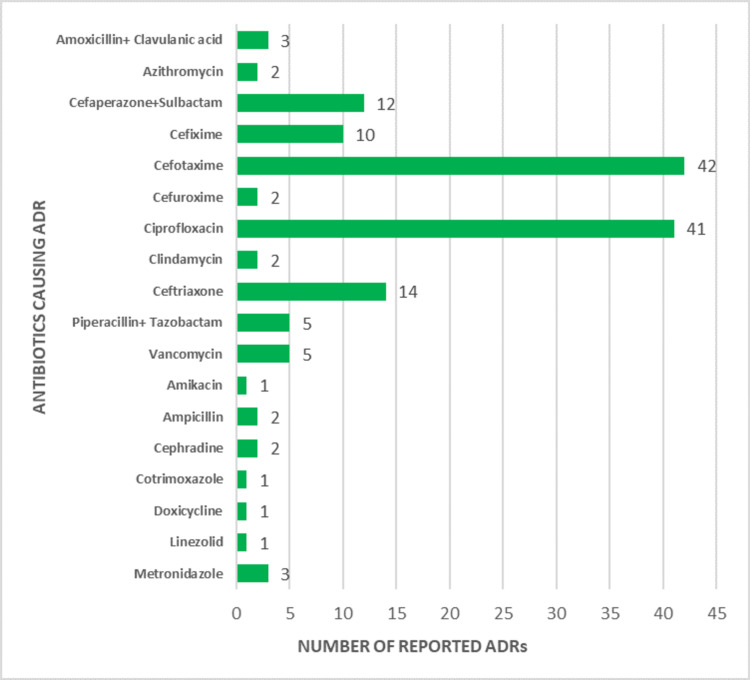
Antibiotics commonly causing ADRs ADRs: adverse drug reactions

Among the antibiotics, cefotaxime was the most common drug reported 42 (28.2%), followed by ciprofloxacin 41 (27.5%), ceftriaxone 14 (9.4%), and cefoperazone + sulbactam 12 (8.1%). The other antibiotics reported were cefixime 10 (6.7%), piperacillin + tazobactam 5(3.4%), vancomycin 5 (3.4%), amoxicillin + potassium clavulanate 3 (2%), and metronidazole 3 (2%). The antibiotics reported to cause ADRs are shown in Figure 3.

**Figure 4:**
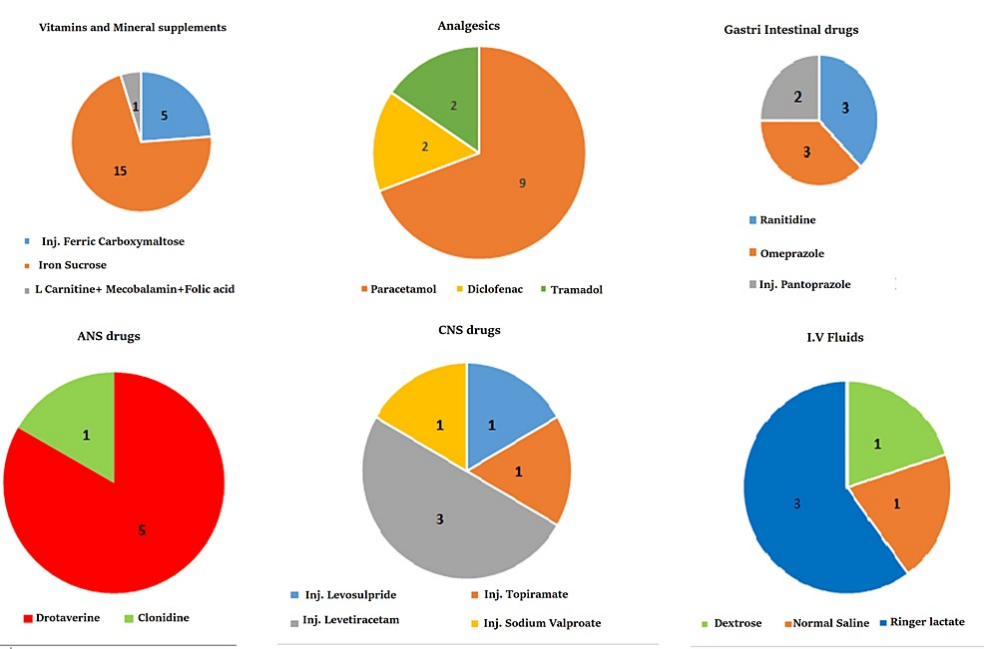
ADRs across drug categories ADRs: adverse drug reaction; ANS drugs: drugs acting on the autonomic nervous system; CNS drugs: drugs acting on the central nervous system; IV fluids: intravenous fluids

The most common vitamins and mineral supplements reported to cause ADRs are iron sucrose 15 (71.4%), ferric carboxymaltose 5 (23.8%), and L carnitine + mecobalamin + folic acid combination 1 (4.8%). Among the contrast dyes, Inj. iohexol is reported to have caused all the adverse effects. The analgesics reported to cause ADRs are paracetamol 9 (69.2%), diclofenac 2 (15.4%), and tramadol 2 (15.4%). Among the GIT drugs, H2 blocker ranitidine 3 (37.5%), ondansetron 3 (37.5%), and pantoprazole 2 (25%) are reported to have caused the adverse effects. Autonomic nervous system (ANS) drugs causing ADRs are drotaverine 5 (83.3%) and clonidine 1 (16.7%). Central nervous system (CNS) drugs reported to cause ADRs are topiramate 1 (16.7%), sodium valproate 1 (16.7%), levosulpiride 1 (16.7%), and levetiracetam 3 (50%). Intravenous (IV) fluids reported were ringer lactate 3(60%), dextrose 1(20%), and normal saline 1(20%). Figure 4 shows the ADRs across drug categories.

**Figure 5:**
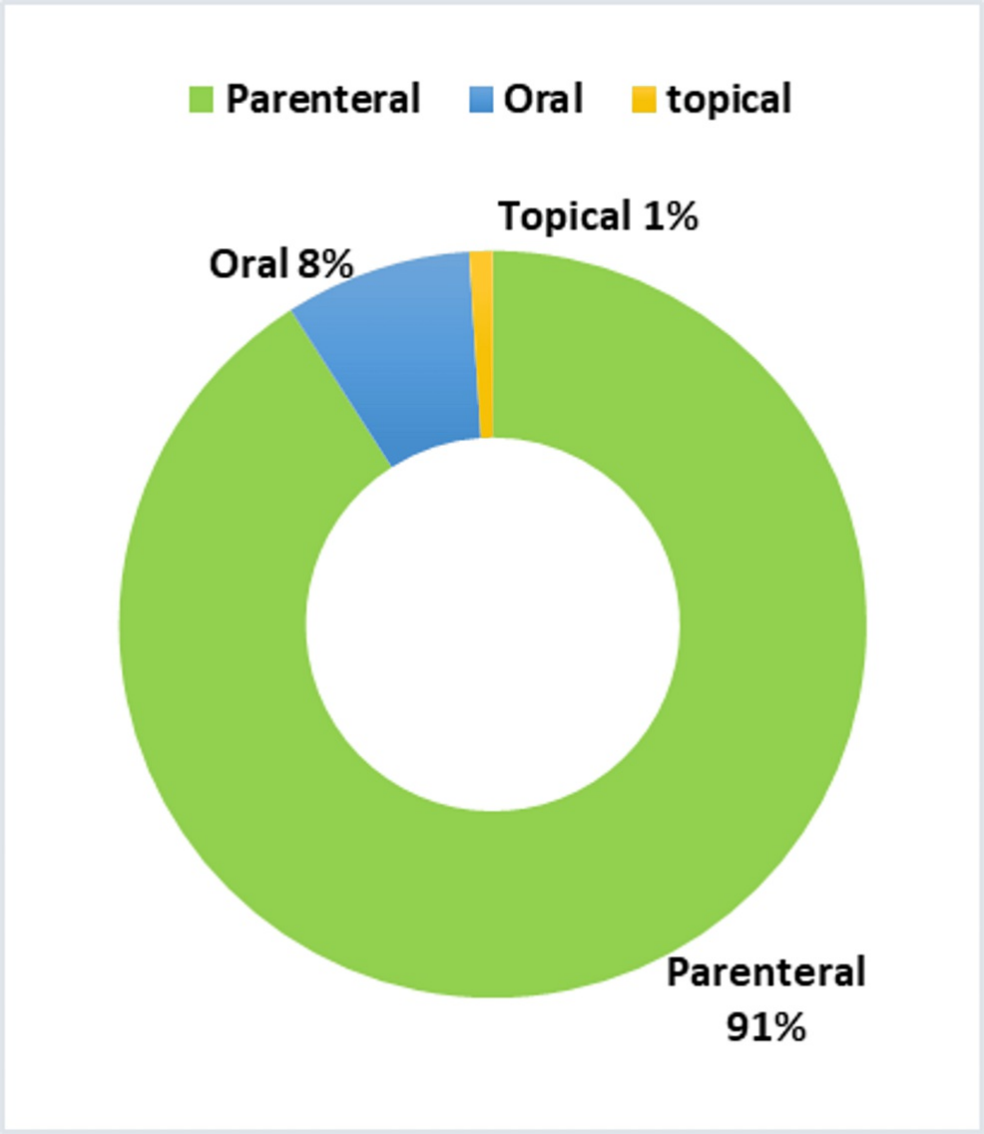
ADRs reported in various drug formulations ADRs: adverse drug reactions

The parenteral route of drug administration reported a maximum number of ADRs of 229 (91%), followed by oral 20(8%) and topical route 3(1%), as shown in Figure 5. The chi-square statistic is 565.75. The p value is <0.00001. The result is significant at p <0.05.

**Figure 6:**
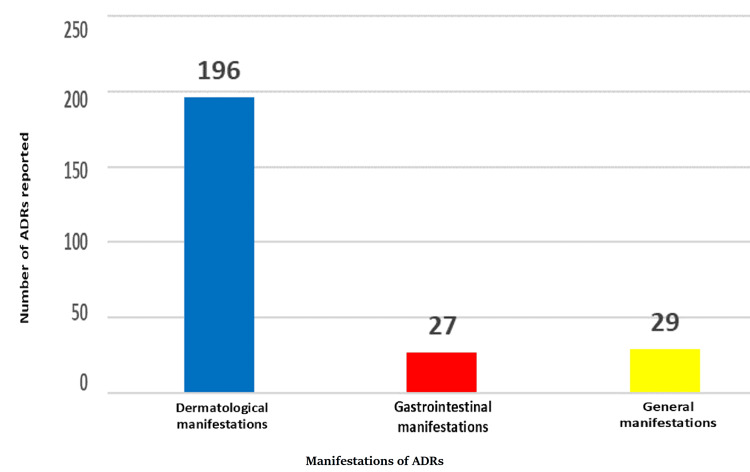
Manifestations of ADRs reported ADRs: adverse drug reactions Dermatological manifestations include rashes, itching, swelling of the injected site, redness, etc. Gastrointestinal manifestations include nausea, vomiting, and diarrhea General manifestations include shivering, tingling, fever, chills, seizures, dyspnea, rigor, and change in blood pressure

Around 196 (77.8%) patients reported dermatological signs and symptoms like rashes, itching, swelling of injected site, redness, etc., 27 (10.7%) of them reported GIT side effects like nausea, vomiting, and diarrhea, and 29 (11.5%) of the population presented with other manifestations like shivering, tingling, fever, chills, seizures, dyspnea, rigor, and change in blood pressure, as shown in Figure 6. The chi-square statistic is 336.0357. The p value is <0.00001. The result is significant at p <0.05.

Based on the Naranjo causality assessment scale, antibiotics, anticonvulsants, and vaccines mostly received a grading of probable for causing ADRs. IV fluids were graded as definite, while analgesics and other medications were deemed to be possible contributors to the adverse reactions. According to the Naranjo causality assessment scaling done, 179 (71%) of the ADRs were categorized as probable, 55 (22%) as possible, 10(4%) as certain, and 8 (3%) as doubtful. The modified Hartwig and Siegel Severity assessment scaling showed 58%, 39%, and 3% of the patients had mild, moderate, and severe ADR, respectively. The causality and severity assessment grading of drugs is represented in Table 3.

**Table 3:** Naranjo causality assessment scaling, and modified Hartwig and Siegel severity assessment of ADRs ADRs: adverse drug reactions ^*^The p value is <0.00001. The result is statistically significant at p < 0.05.

Categories		Number	Percentage (%)	Chi-square value	P value
Causality grading	Probable	179	71	409.6085	<0.00001^*^
	Possible	55	22		
	Certain	10	4		
	Doubtful	8	3		
Severity grading	Mild	146	58	175.2857	<0.00001^*^
	Moderate	98	39		
	Severe	8	3		

**Figure 7:**
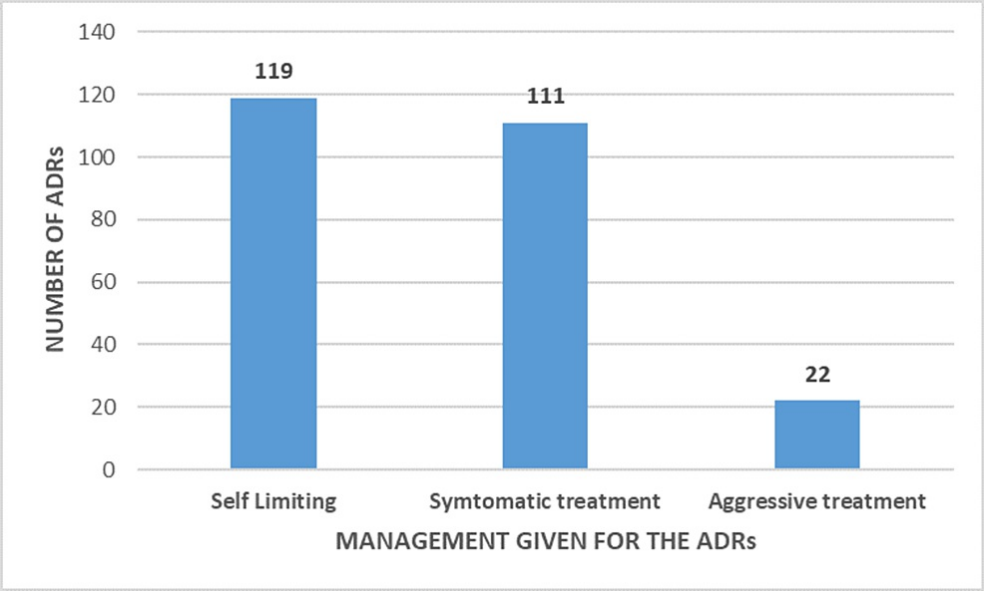
Management of ADRs ADRs: adverse drug reactions Self-limiting includes ADRs that do not require any treatment Symptomatic treatment encompasses interventions aimed at managing the symptoms of ADRs, necessitating specific attention and care to address the effects of such reactions Aggressive treatment encompasses situations where ADRs lead to prolonged hospital stays, necessitate admission to the intensive care unit, or require initial hospitalization specifically due to the encountered ADRs

Almost 119 (47.2%) of the ADRs were self-limiting and did not require treatment, 111 (44.1%) of the reactions required symptomatic treatment, and 22 (8.7%) of the reactions needed aggressive treatment for the ADR with prolongation of hospital stay, admission in intensive care unit for the ADR, or initial hospitalization for the ADR encountered, as shown in Figure 7. The chi-square statistic is 103.5357. The p value is <0.00001. The result is significant at p <0.05.

## Discussion

In our study, all 252 ADRs were reported on the CDSCO suspected ADR reporting form. There were 54 ADRs reported in both 2019 and 2022. In 2020 and 2021, there were notably fewer ADRs reported, with 33 and 22 cases, respectively, possibly influenced by the circumstances surrounding the COVID-19 pandemic. In 2023, a maximum of 77 ADRs were reported due to the implementation of frequent sensitization and awareness programs on ADR reporting within our hospital. Due to a limited observation period of two months in 2024, only 12 ADR cases were reported.

During the study period, no statistically significant difference existed between the number of ADRs reported in male and female cases. The susceptibility to ADRs is commonly perceived to be higher in vulnerable age groups, according to several studies compared to middle-aged individuals [12-14]. However, our study presents a different perspective on this matter. The most commonly affected age group was 20-40 years, consistent with findings from a study by Rajeshreddy et al., where higher numbers of ADRs were reported in the 21-30 and 31-40 age groups [15]. The higher occurrence of ADRs in this age group may be attributed to their more frequent visits to hospitals, driven by several factors. These include a priority on health maintenance through regular checkups and preventive care measures, management of chronic conditions like diabetes and hypertension, seeking reproductive health services such as gynecological care and family planning, encountering emerging health issues related to lifestyle choices, navigating significant life transitions such as career changes and starting families, benefiting from easier access to healthcare through insurance or public programs, and demonstrating increased health consciousness by seeking advice on diet, exercise, and overall wellness trends.

The distribution of ADRs across different hospital departments can be attributed to various factors within each department. The Obstetrics and Gynecology Department often administers medications for prenatal care, labor, and postpartum management due to dealing with pregnant women and women's health issues, potentially leading to a higher incidence of adverse reactions. General surgery patients frequently receive medications for pain management, infection prevention, and anesthesia during surgical procedures, contributing to a higher likelihood of adverse reactions. General medicine departments, handling a wide range of medical conditions like diabetes and hypertension, may witness more adverse reactions due to the diverse medication requirements. Pediatrics, dealing with children who are more susceptible to adverse reactions and requiring medications for various conditions, also contributes to the occurrence of adverse reactions. In emergency medicine, where medications are rapidly administered in acute cases with incomplete medical histories, the risk of adverse reactions is increased. These department-specific factors collectively contribute to the observed distribution of ADRs in hospital settings.

Around 32% of the reactions were type A, and 68% were type B. Hence, most of the reactions reported in our study were unexpected or bizarre and highly unpredictable. This finding contrasts the general overall incidence of type B adverse reactions, which comprise only 10-15% of reported ADRs worldwide [16]. Hence, this shows that clinicians and healthcare workers in our hospital are highly underreporting the type A reactions and stressing only the uncommon type B reactions. Physicians should also be trained and made aware of the importance of reporting expected ADRs.

The predominance of ADRs caused by antibiotics, followed by vitamins and mineral supplements, contrast dyes, anti-TB drugs, analgesics, and GIT drugs in decreasing order, can be explained by several factors: antibiotics are the most common cause of adverse reactions, accounting for 59.1% of cases due to their widespread use in treating bacterial infections and the complexity of their mechanisms and side effects. Factors such as overuse, high doses, and patient sensitivities contribute to their prevalence. This is in accordance with the study conducted by Badyal et al. [17], Shareef et al. [18], and Giardina et al. [19]. Among the antibiotics, cefotaxime emerges as the most commonly reported drug, accounting for 28.2% of cases, followed closely by ciprofloxacin at 27.5%. Other notable antibiotics include ceftriaxone at 9.4%, cefoperazone + sulbactam at 8.1%, and cefixime at 6.7%. Additional antibiotics reported include piperacillin + tazobactam, vancomycin, amoxicillin + potassium clavulanate, and metronidazole. This distribution reflects the widespread use of these antibiotics in clinical practice, potentially increasing the likelihood of ADRs associated with their use.

Vitamins and mineral supplements, although generally safe, can lead to adverse reactions, particularly with excessive intake or interactions with other medications. The prevalence of ADRs linked to iron sucrose, ferric carboxymaltose, and L-carnitine + mecobalamin + folic acid combinations in vitamins and mineral supplements, with iron sucrose being the most commonly reported, can be attributed to various factors such as individual patient sensitivities, dosing regimens, and underlying health conditions. Similarly, the exclusive reporting of adverse effects associated with Inj. Iohexol among contrast dyes may be due to its widespread use in diagnostic imaging procedures, potentially leading to a higher likelihood of adverse reactions being observed and reported. Anti-TB drugs, vital for TB treatment, are associated with notable side effects like hepatotoxicity, GIT disturbances, and hypersensitivity reactions. Their formulation as a multidrug regimen in fixed doses heightens the risk of ADRs.

Analgesics, typically administered for pain management, possess the potential to induce adverse reactions such as GIT bleeding, renal impairment, and allergic responses. The reporting of ADRs linked to paracetamol, diclofenac, and tramadol indicates differing levels of associated risks. Paracetamol, the most frequently reported analgesic, likely owes its prevalence to its widespread use for pain alleviation and fever reduction. While diclofenac and tramadol are less commonly cited, they remain widely prescribed analgesics, each carrying distinct risk profiles for adverse reactions. The underlying causes of these ADRs may include individual patient susceptibilities, treatment regimens, and existing health conditions.

GIT drugs, essential for managing GIT disorders, can induce adverse reactions like nausea, diarrhea, and constipation. In our study, antiulcer drugs such as ranitidine and omeprazole were found to cause a significant number of ADRs. This could be attributed to the widespread administration of these drugs to nearly all patients admitted to our hospital, regardless of their department of admission. Consequently, the increased exposure to these medications increases the likelihood of ADRs.

The reported ADRs associated with drotaverine and clonidine, both acting on the ANS, indicate a higher occurrence of adverse effects with drotaverine. This could be due to factors such as its widespread use and potential for impacting various physiological systems within the ANS. Among CNS drugs, the ADRs reported with topiramate, sodium valproate, levosulpiride, and levetiracetam suggest varying degrees of risk associated with each medication. The reported adverse reactions linked to IV fluids such as Ringer's lactate, dextrose, and normal saline may be influenced by factors like the frequency and volume of administration, patient characteristics, and underlying medical conditions. The prevalence of ADRs across these groups reflects their widespread use, complexity, and potential for causing various side effects.

Most of the ADRs reported are due to the parenteral route of drug administration, followed by the oral and topical routes. This is similar to the study conducted by Sen et al. [20]. This could be due to the direct delivery of the drug into systemic circulation by the first-pass metabolism and its immediate effect. To some extent, this can be prevented by giving a test dose of the drug prior to the therapeutic dose needed.

Around 77.8% of patients reported dermatological signs and symptoms like rashes, itching, swelling of the injected site, and redness, 10.7% of them reported GIT side effects like nausea, vomiting, and diarrhea, and 11.5% of the population presented with fever, chills, and a change in blood pressure. The results were significant, with a p value of <0.05. The increased incidence of dermatological manifestations aligns with findings from studies by Rana et al. [21], Shrivastava et al. [22], and Chan et al. [23], indicating consistency across different research contexts. Patients' heightened awareness of visible ADRs impacting the skin could lead them to seek medical assistance at hospitals promptly. Similarly, concerns regarding GIT symptoms arising from medication usage are common among individuals. Nonetheless, it is essential to acknowledge that widely prescribed medications like antimicrobials, vitamins and mineral supplements, and IV fluids are prone to causing ADRs despite their extensive use. These skin and GIT adverse reactions may worsen the quality of life and increase health expenditure and hospitalization.

According to the Naranjo causality assessment scale, 77% of the ADRs were categorized as probable, 22% as possible, 4% as certain, and 3% as doubtful. The number of reactions classified into probable categories is similar to other studies conducted previously by Gupta and Kumar [24], Ramasubbu et al. [25], and Patel et al. [26]. In our study, ADRs due to IV fluids were classified as certain as all the reactions occurred immediately after drug administration without any confounding factor. ADRs due to other drugs like antibiotics, analgesics, and anticonvulsants fall into the possible or probable category due to the intake of other drugs along with the suspected drug, the underlying disease condition, a lack of dechallenge and rechallenge information, etc.

According to the modified Hartwig and Siegel severity assessment scale, 58%, 39%, and 3% of the patients had mild, moderate, and severe ADRs, respectively. No deaths due to ADRs were reported in our hospital. These findings are similar to the study conducted by Rajeshreddy et al. [15], Kharb et al. [27], and Ponnusankar et al. [28]. Most of the ADRs reported were self-limiting or required minor symptomatic treatment. This is similar to the study conducted by Badyal et al. [17]. However, few patients required intensive management and prolongation of hospital stay due to the ADR. These reactions were, however, expected to be type B reactions. Enquiring about the history of drug allergies, administration of prior test doses, awareness regarding ADRs, and their management among medical practitioners can significantly reduce the severity of these ADRs, though they are inevitable.

A notable advantage of our study is its retrospective design, covering a duration of five years. While there are numerous studies on similar topics, only a few have the same extensive timeframe, thus setting our research apart and offering valuable insights over an extended period. A significant constraint of our study was the inability to conduct logistic regression analysis due to the absence of data on the total number of cases visiting our hospital throughout the study period. This limitation stemmed from the lack of a comprehensive database capturing patient attendance and demographic details during the specified timeframe. Another notable limitation of our study is the limited number of reported ADR cases, which diminishes the reliability of our findings and precludes robust statistical analysis. The low reporting of ADRs in our study may be due to unintentional oversight, potentially stemming from work-related stress and forgetfulness. Additionally, inadequate awareness of the significance of drug safety monitoring, a limited understanding of ADR reporting program objectives, and the bustling nature of outpatient settings could contribute to underreporting. Furthermore, for many clinicians, reporting ADRs may not be perceived as a prioritized task. Hence, there is a need to enhance ADR monitoring in this diverse region by raising awareness and motivating healthcare providers to report ADRs actively.

## Conclusions

The observed pattern of ADRs in our hospital aligns with findings from other studies, indicating a consistent trend across different healthcare settings. While the majority of reported reactions are unpredictable and relatively mild, their documentation underscores the importance of raising awareness among clinicians and regulatory authorities. This heightened awareness is crucial for ensuring the safe use of medications and preventing potential near-misses that could escalate into serious consequences. Our study serves as a valuable database for recognizing, documenting, and mitigating preventable ADRs. By providing comprehensive insights into the occurrence and characteristics of ADRs, it empowers healthcare practitioners to identify and report adverse events more effectively. This, in turn, enhances the ability of healthcare teams to manage ADRs efficiently and implement targeted interventions to improve patient safety and medication management practices.
